# The economy of social resources and its influence on spatial perceptions

**DOI:** 10.3389/fnhum.2013.00772

**Published:** 2013-11-19

**Authors:** Elizabeth B. Gross, Dennis Proffitt

**Affiliations:** Department of Psychology, University of Virginia, CharlottesvilleVA, USA

**Keywords:** spatial perception, social baseline, social resources, visual perception, extraversion, attachment style

## Abstract

Survival for any organism, including people, is a matter of resource management. To ensure survival, people necessarily budget their resources. Spatial perceptions contribute to resource budgeting by scaling the environment to an individual’s available resources. Effective budgeting requires setting a balance of income and expenditures around some baseline value. For social resources, this baseline assumes that the individuals are embedded in their social network. A review of the literature supports the proposal that our visual perceptions vary based on the implicit budgeting of physical and social resources, where social resources, as they fluctuate relative to a baseline, can directly alter our visual perceptions.

## The economy of social resources and its influence on spatial perceptions

Conscious visual experience suggests that our perceptions simply mirror the environment as it is. However, visual perception varies with changes in our physical and social environments, which suggests that our visual system does not provide a geometrically accurate representation of our world, but rather one that is grounded in action capabilities and social influences (Proffitt and Linkenauger, 2013). Both physiological resources, for example, blood glucose, and social resources, such as supportive friends, can influence perceptions of the spatial environment (Proffitt, 2006; Schnall et al., 2008). Focusing on social influences on perception, the current paper offers the hypothesis that the magnitude of social resources is evaluated with respect to a baseline, and that visual perception will reflect variations around this baseline.

Research in behavioral ecology suggests cost-benefit resource analyses predict individual behavior. In order to determine costs and benefits in an economy of action, resources must be evaluated relative to a baseline, defined as a value around which costs and benefits are balanced. In the current paper, we review evidence that suggests our visual perceptions are scaled to both our physiological and social resources. Next, this paper introduces the concept of a social baseline as a reference value for evaluating social resources. Social Baseline Theory (SBT), introduced by Coan, Beckes, and colleagues (Beckes and Coan, 2011, 2012; Coan et al., in press), serves as a useful framework within which to construe social resources. We propose that a person’s social baseline is set by the quality and breadth of their social network. Moreover, individual differences in attachment style and personality produce a unique social baseline for each individual. Variability in social environments will interact with the individual’s baseline to produce fluctuations in social resources. This flux of social resources relative to a baseline will produce corresponding changes in visual perception. Finally, we discuss how changes in the social environment also interact with individual baselines to produce changes in visual perception. This resource budgeting account derives, in part, from considerations of behavioral ecology and human physiology.

In order to evaluate and budget for the potential resource costs and benefits of an action, it is necessary to first determine a baseline, or the amount of resources the body will seek to maintain. Like a household budget, there is typically some desired positive value of savings around which income and expenditures are balanced. Rather than spending all of your income, some amount of monetary resources is protected. When the amount of savings dips below the baseline, resources are conserved by cutting unnecessary expenditures until the savings are restored. Alternatively, when the savings value is higher than the baseline, expenditures might increase.

The concept of a baseline in an economy of action is omnipresent in human physiology, for example in the maintenance of bodily glucose levels. There are multiple sources of energy in the body, but glucose, which exists as both blood glucose and as glycogen stores in the muscles and liver, is the main energy source for both our muscles and the brain (Benton et al., 1996). When blood glucose levels decline, glycogen stores are released into the bloodstream to restore glucose levels to baseline; likewise, when blood glucose levels rise above a baseline level, insulin is released and blood glucose is transported to and stored in the muscles and liver as glycogen (Benton et al., 1996). In other words, there exists a baseline level of blood glucose and, barring any medical disorders, the human body seeks to conserve this baseline, much like a thermostat.

For all animals, survival is a matter of budgeting physiological resources, where at the most basic level animals ultimately must not expend more calories than they consume. The field of behavioral ecology elegantly demonstrates how animal behavior is predicted by models that optimize the cost-benefit ratio inherent in their actions. For example, eating larger prey means a higher caloric gain for a predator; however, eating bigger prey may also engender a higher cost. Shore crabs are sensitive to the cost-benefit ratio of prey size, and when shore crabs are given the opportunity to eat mussels of all sizes, their diet consists mostly of mussels affording the highest rate of caloric intake, not the largest mussels with the highest caloric value but hardest to crack open shells (Elner and Hughes, 1978). Such findings are abundant in the field of behavioral ecology, and they suggest that animals are sensitive to both the costs and benefits of their actions.

Cost-benefit analyses are also evident in visual perceptions. Research has shown that the visual system is sensitive to the costs and benefits of individuals’ actions with respect to their bodily resources and social environments (Proffitt, 2006). One of the first studies to show a role of bioenergetics resources in spatial perception did so in the context of viewing hills (Proffitt et al., 1995). In virtually all circumstances, individuals overestimate the slants of hills. One striking anecdote of this phenomenon is to consider the streets in San Francisco. Even in pictures, these streets appear to be astronomically steep, but the steepest street in San Francisco is reportedly 17.5 degrees (Naylor and McBeath, 2008). The general overestimation of geographical slant was originally reported in the literature by Kammann (1967), but more recently it has been systematically studied by Proffitt and colleagues (Proffitt et al., 1995). They found that participants overestimated the slant of a 10 degree hill to be approximately 30 degrees when standing at the bottom of the hill (Proffitt et al., 1995), and the effect persists even when participants are allowed to view a cross-section of the hill (Proffitt et al., 2001). More importantly, participants who are physically fatigued, elderly, not physically fit, or encumbered with a heavy backpack^1^ estimate the slant of a hill to be steeper than their counterparts, suggesting that perception varies with the effort and ability required to perform an action (Bhalla and Proffitt, 1999; Proffitt, 2006).

Research has found a direct physiological basis for overestimations in visual perception. Schnall et al. (2010) found that participants, who consumed a caloric drink that restored blood glucose levels following a cognitively depleting task, estimated the slant of a hill to be significantly less steep than those who had consumed a no-calorie drink after the depletion task. Additionally, Zadra et al. (2010) demonstrated that direct physiological measures of individual fitness predicted distance perception, in particular maximal aerobic capacity (VO2) max at blood lactate threshold (the gold standard measure of physical fitness). Those who were more fit perceived targets as being closer than those who were less fit. These findings suggest that perception is influenced by the bioenergetic costs of acting on an extent relative to the amount of physical resources available in the body.

Moreover, research suggests visual perception is sensitive to *anticipated* resources and costs. Thirsty participants perceived a bottle of water to be closer than non-thirsty participants (Balcetis and Dunning, 2010), and participants engaged in dieting perceived muffins to be larger in size than non-dieters (Van Koningsgruggen et al., 2011). Additionally, participants report that threatening objects, such as spiders, appear to be closer, larger (Vasey et al., 2012), and moving faster (Witt and Sugovic, 2013) than non-threatening objects. These findings suggest that perception also varies with motivations to acquire physiological resources and avoid threatening objects (Dunning and Balcetis, 2013; Riccio et al., 2013), presumably to facilitate acting on the environment (Witt and Sugovic, 2013). Collectively, the above studies demonstrate that the visual system also includes potential environmental benefits and costs in a cost-benefit analysis of resources.

Physical resources are not the only resources that people have at their disposal. As humans, we do not behave in isolation; rather, we function embedded in a social environment. People’s ability to act in the environment is augmented if they have a friend or family member who will act on their behalf. Given that physiological potential influences perception, then the availability of social support provided by others should also influence visual perception. Indeed, there is evidence to support this claim. Schnall and colleagues (Schnall et al., 2008) demonstrated that participants who were either walking with or imagining a supportive friend gave lower slant estimates than participants who were walking alone or imagining a non-supportive friend. This has recently been extended to online social networking, where participants who browsed the Facebook profile of a supportive friend estimated the slope of a hill to be less steep than those who browsed the profile of a non-supportive friend (Faulkner and Clore, 2012). In an attempt to understand the mechanisms by which friends are influencing visual perception, Oishi et al. (2013) manipulated felt understanding between strangers and found that the participants who believed that the other participant understood their personality perceived a hill to be less steep than those who believed they were not understood.

Potential social costs also influence perception. Participants perceive aggressive male students to be standing closer than non-aggressive males (Cole et al., 2013) and threatening out-group members are perceived to be closer than non-threatening out-group members (Xiao and Bavel, 2012). Additionally, social resources can attenuate the effect of social costs. Following social rejection, participants report the interpersonal distance to accepting others to be closer than rejecting others (Knowles et al., 2013), and Harber and colleagues (Harber et al., 2011) report that psychosocial resources, such as self-worth, reduced perceived distance to threatening objects. Collectively, these findings indicate that social resources can function in a similar fashion to physiological resources, where social costs and benefits work to influence visual perception.

Coan and colleagues propose that “load sharing” is the mechanism by which social resources alter cognitive processes (Coan et al., in press). To successfully act in the environment, individuals must identify and solve a set number of problems. The social network allows individuals to offload problems, effectively reducing the cost of acting. An example from behavioral ecology clearly illustrates this mechanism. When feeding, ostriches must simultaneously hunt for food and avoid predators. Hunting in groups allows the ostrich to offload the work of scanning for predators, resulting in more time to consume food than when feeding alone (Bertram, 1980). For the ostrich, hunting in groups does not increase the amount of available food, in fact it reduces it; rather, it increases the time spent foraging which more than offsets the cost of competing with others in the group. Similarly, in humans it is not that the presence of social support indicates a greater quantity of tangible resources. Instead, social support signifies the ability to offload work to the social network, which reduces the overall cost of acting in the environment.

The aforementioned principles regarding costs and benefits relative to a baseline value are applicable to a variety of ecological environments, including our social environment. Again, there exists research that suggests that, much like physical resources, our visual perceptions vary with changes in the social environment (Schnall et al., 2008; Harber et al., 2011; Faulkner and Clore, 2012; Knowles et al., 2013; Oishi et al., 2013). While there is an extensive literature on how costs and benefits are evaluated and maximized in human physiology, there is considerably less research investigating how social resources are evaluated. However, there is evidence that the concept of a baseline is paramount to evaluating social resources. In the social support literature, not receiving social support is most detrimental when support was expected, and receiving unexpected social support is more beneficial than receiving expected social support (Bergeman et al., 2010). That is, the costs and benefits of social support are evaluated relative to baseline expectations. What remains, then, is to define and determine the components that set the expected social baseline with which we evaluate our social resources.

One idea in particular, aptly named SBT, addresses this issue (Beckes and Coan, 2011, 2012; Coan et al., in press). For much of psychology, the unit of analysis is focused solely on the individual; the assumption being that the presence of social support adds resources to an otherwise self-sufficient individual. SBT asserts that the individual’s default state is to assume social support. In other words, an individual’s social baseline, by which an environment is determined to be costly or beneficial, includes the individual and part of their social network (Beckes and Coan, 2011). As social animals, people assume the presence of social support, which decreases the cost of acting by load sharing (Coan et al., in press). A person’s social baseline assumes the presence of social support, and thus, to study an individual in isolation is to study someone whose resources are taxed.

However, just as variability exists in physiology across individuals, there exist differences in the social baselines of individuals. While almost all people function embedded in a social network, individuals will differ in the amount and quality of anticipated social resources. For the remainder of the paper, our attention turns to a discussion of the possible individual and situational differences that will interact to influence an individual’s sense of social support. Based on the existing literature, we propose that individual differences, such as attachment style and personality traits, can set an individual’s social baseline. Additionally, the state of the social network itself can vary. Differences that exist outside of the individual, for example the action capabilities of the friends within the network, can cause variations that interact with the baseline of social support. Ultimately, we propose that these individual differences in social resources and the social environment should be reflected in visual perception.

SBT proposes that the individual’s baseline resources are composed of both their own resources and those in their social network. We propose that social baselines vary across individuals and are determined, in part, by our early life experiences. In biology, studies in life history theory show that, across a wide range of organisms, nutritional deficits early in life are followed by an initial compensation that results in costly deficits later in life (Metcalfe and Monaghan, 2001).Variability in early life changes the organism’s baseline to be lower such that, over time, they will show nutritional and growth deficits.

Similarly, in attachment style theory, variability in early life experiences in caregiver relationships will affect an individual’s relationship styles well into adulthood (Bowlby, 1969). Children whose caregivers were attentive and responsive to their needs will develop a secure attachment style; they are comfortable and confident in their current relationships. On the other hand, if a child’s primary caregiver responded inconsistently, the child will often develop an insecure or anxious attachment style. Insecurely attached individuals are concerned about the reliability and dependability of their current relationships (Ainsworth et al., 1978; Bartholomew and Horowitz, 1991). Similar to findings in biology, variability in early life relationships will negatively affect an individual’s relationships over their lifetime.

The impact of attachment style is far reaching; attachment style also moderates the benefits of social support such that insecurely and anxiously attached individuals report less perceived social support; anxiously attached participants perceive supportive messages from their romantic partners to be less supportive (Collins and Feeney, 2004), and securely attached individuals that spent time in the presence of their romantic partners before a social stress task reported lower state anxiety levels than insecurely attached individuals (Ditzen et al., 2008). In sum, individuals that are more anxious about their relationships perceive that they have fewer social resources, and they benefit less from received social support. Presumably, these individuals regard supportive others as less reliable, rendering them unable to invest wholeheartedly in their social network.

A social baseline indicates the degree to which an individual incorporates others in their network of social resources. Individuals with a lower social baseline are more autonomous, meaning they are less likely to incorporate others as part of their resource pool. This value is independent of whether or not the individuals in their social network engender resources or costs. We propose that insecure and anxiously attached individuals’ social baselines are set to a lower value. As a consequence, if the individuals that comprise a social network are particularly supportive, then insecurely and anxiously attached individuals will be less likely to utilize available social resources, a claim that is supported by research discussed above (Collins and Feeney, 2004; Ditzen et al., 2008). However, social relationships are dynamic, and at times the social network requires individuals to return a favor. In the instances where the social network is imposing a burden on the individual, anxiously and insecurely attached participants should be less burdened. That is, with a lower social baseline (indicating more autonomy), an individual is also less likely to include the burdens of their social network into the total calculation of their costs.

In addition to attachment style, we expect that social baselines will also vary with individual differences in extraversion. According to Eysenck’s personality theory, differences in arousal levels lead extraverts to seek out social contact and introverts to avoid social contact (Matthews and Gilliland, 1999). As a result, extraverts tend to have a larger social support network (Stokes, 1985; Cohen et al., 1997; Swickert et al., 2002) and report interacting more often with their social support network (Swickert et al., 2002). Additionally, extraverts are more likely to seek out social support (Amirkhan et al., 1995; Halamandaris and Power, 1999) and report more perceived and enacted social support than introverts (Swickert et al., 2002, 2010). Overall, extraverts report having more social resources and benefit more from social support, suggesting they are more inter-dependent; they may be more likely to include others’ resources into their implicit assessment of their own costs and benefits. As such, we propose extraverts have a higher social baseline than introverts. Because the default state is to expect the support of a social network, extraverts have more assumed resources at their baseline than introverts. Of importance to note is that, due to their higher social baseline, extraverts incur more of a cost than introverts when they are called upon to support their social network.

Thus far, we have discussed the individual differences expected to produce higher or lower social baselines, namely, attachment style and extraversion. Another source of variability in social resources arises from the social network itself. As previously mentioned, those in the social network could either be an available resource or, depending on their capabilities, an added burden. For illustrative purposes, consider moving into a new apartment with a friend. Typically, the friend would share the load of carrying heavy boxes, rendering her a potential resource. However, suppose the friend has recently broken her leg. Now you are responsible for moving all of your and her personal belongings; your friend is now an added cost. The social baseline has remained the same, it includes your friend, but situational factors have drastically changed the impact on expected costs and benefits. In fact, altering the action capabilities of friends has been shown to mediate the effect of social support in visual perception. In a study by Doerrfeld et al. (2012), participants estimated the weight of boxes to be less heavy if a friend was helping, but not when the friend was present but physically impaired. In another study, participants playing pong estimated the speed of the ball to be traveling faster when it was more difficult for their partner to block the ball (Witt et al., 2012). As this research demonstrates, the capabilities of the social network are an important point to consider. With respect to SBT, it highlights that higher social baselines are not always better. Higher social baselines indicate that you are also more likely to incorporate the burdens of the network, resulting in times where a higher baseline results in an added cost. Therefore, the amount of total available social resources depends on both the social baseline in addition to the quality and capabilities of the social network itself.

The proposed conceptualization of social resources has several implications for visual perception. Our visual perceptions are scaled to our physiological and social resources; as we accrue resources, distances appear closer and slants appear to be less steep, and vice versa (Proffitt, 2006; Schnall et al., 2008; Zadra et al., 2010). Social resources are evaluated relative to the individual’s baseline, an indicator of the degree to which an individual includes others in their social network and the quality of the social relationship. When the social network is a resource, individuals with a higher baseline are more likely to include others as part of their evaluation of resources. In this case, individuals with a higher social baseline should perceive distances to be closer and slants to be less steep. Alternatively, when the social network is a burden, individuals with a higher social baseline will have an increase in their social costs, and their visual perceptions will reflect this increase such that distances appear to be farther and hills appear to be steeper. We propose that social baselines are determined, in part, by individual differences such as attachment style and extraversion. Extraverts and securely attached individuals have a higher social baseline compared to introverts and insecurely attached individuals. As a result, extraverts and securely attached individuals should perceive hills to be less steep and distances to appear closer relative to their peers, except when the social network is a burden. In that case, extraverts and securely attached individuals should perceive distances to be farther and hills to be less steep. In sum, the individual differences that reflect changes in the social baseline should also interact with the social network to produce changes in visual perceptions.

In conclusion, people adapt to and attempt to thrive in both social and physical environments, and studying individuals in isolation ignores a vital component of humans’ ecological environment. Still, it is not simply that the presence of a friend is a guarantee of social resources. We propose social resources are evaluated in accordance with a baseline that varies with individual differences and with respect to the capabilities of the social network. Our visual perceptions reflect the implicit budgeting of physical and social resources. For social resources, fluctuations around the social baseline and variations in the state of the social network will cause corresponding changes in visual perception. Ultimately, this proposal prompts researchers to consider a more nuanced study of how social environments differentially impact visual perception.

## Conflict of interest statement

The authors declare that the research was conducted in the absence of any commercial or financial relationships that could be construed as a potential conflict of interest.

## Footnotes

^1^There is criticism that the increase in slant estimates while wearing a heavy backpack is due to experimental demand characteristics (Durgin et al., 2010). While this is certainly a possibility, we do not feel that the support for this claim is convincing (Proffitt and Zadra, 2011).
